# Associations between Red Meat Intakes and the Micronutrient Intake and Status of UK Females: A Secondary Analysis of the UK National Diet and Nutrition Survey

**DOI:** 10.3390/nu9070768

**Published:** 2017-07-18

**Authors:** Emma Derbyshire

**Affiliations:** Nutritional Insight Limited, Surrey KT17 2AA, UK; emma@nutritional-insight.co.uk; Tel.: +44-(0)-758-437-5246

**Keywords:** red meat, iron, zinc, vitamin D, women’s health, dietary guidelines

## Abstract

Blanket health messages to lower red meat intakes are being communicated at present. These could have adverse implications on the micronutrient quality of women’s diets. The current paper evaluates the nutritional impact of lower red meat intakes on British women’s micronutrient intakes and status. A secondary analysis of the UK National Diet and Nutrition Survey was undertaken using data from years 2008/2009 to 2011/2012. This was comprised of dietary and blood analyte data from 1384 and 641 females aged 11 to 64 years. Females consuming less than 40 g total red meat daily were more likely to have micronutrient intakes below the Lower Reference Nutrient Intake (LRNI) for zinc, iron, vitamin B12 and potassium and have lower habitual vitamin D intakes than females consuming between 40 and 69 g daily. After adjusting data for energy intake, zinc (% below the LRNI) and vitamin D (μg/day) remained statistically significant (*p* < 0.001). No significant differences were observed for blood biomarkers. Females consuming diets lower in red meat, i.e., <40 g daily, appear to have reduced micronutrient intakes, especially in the case of zinc and vitamin D. This should be considered when giving blanket advice for whole populations to reduce red meat intakes.

## 1. Introduction

Relative to its energy profile, the nutritional quality that red meat provides and its contribution to key micronutrients is often under-valued [1]. Findings from the latest UK National Diet and Nutrition Survey (NDNS) highlight sizeable gaps in habitual meat intakes between UK males and females [2]. Reported mean intakes of red and processed red meat from Years 5 and 6 of the survey were 47 g per day for females aged 19 to 64 years. These intakes were significantly lower than the 58 g per day reported intake for years 1 and 2 (combined). This indicates a downward trend over a 4-year period amongst UK women [2] which appears to be a reflection of long-term reductions in red and processed meat intake that is occurring in many high-income countries.

These intakes are also substantially lower than Scientific Advisory Committee on Nutrition advice which was for those eating more than 90 g of red and processed meat daily (cooked weight) to aim for 70 g per day [3]. These guidelines were also based on the assumption that this level of intake would have little effect on the proportion of UK adults with iron intakes below the Lower Reference Nutrient Intake (LRNI) [3]. Modelling exercises within the report also identified that red and processed red meat made a greater contribution to female’s zinc intakes than iron intakes. Subsequently, it was proposed that reducing meat intakes amongst those with already lower intakes could drive up the risk of iron and zinc deficiency [3].

It is well appreciated that females are at particularly high risk of iron deficiency (ID) and iron deficiency anaemia (IDA). ID is characterised by diminished iron stores whilst IDA is attributed to the combined effects of poor iron stores and diminished haemoglobin levels [4]. Menstruation, blood donation, diets of low iron bioavailability and the physiological demands of pregnancy all take their toll on female’s iron stores [5]. Iron is also critical to reproductive health, with IDA having wide-ranging detrimental effects on maternal and infant well-being [6]. Some research has also linked ID in females of childbearing age to reduced cognition, mental health and heightened fatigue [7].

With regard to zinc, the human genome encodes around 3000 different zinc proteins, each playing key roles at the cellular level, with the impact of zinc on health and disease thought to be as far-reaching as iron [8]. Zinc has also been demonstrated to have stimulatory effect on osteoblastic bone formation and mineralization, stimulating aminoacyl-tRNA synthetase and cellular protein synthesis, indicating an important role on women’s bone health [9]. In a more general sense, zinc also modulates cell-mediated immunity and acts as an antioxidant and anti-inflammatory agent [10].

Alongside iron and zinc, lean red meat is also an excellent provider of vitamin B_12_, niacin, vitamin B_6_, phosphorous and source of riboflavin, pantothenic acid, selenium and some vitamin D [11]. Touching on the roles of these, B-vitamins perform essential roles in cell function, acting as co-enzymes and having potential roles in brain health, possibly via the synthesis of neurochemical and signalling molecules [12]. B-vitamins may also provide protection against osteoporosis, especially amongst those with malabsorption conditions such as coeliac disease [13]. With regard to dietary selenium, this acts essentially through selenoproteins which are involved in diverse cellular functions [14]. Whilst continued intervention trials are needed, selenium deficiency has been linked to female infertility, miscarriage, preeclampsia, gestational diabetes, foetal growth restriction, preterm labour and obstetric cholestasis [15]. Suboptimal vitamin D status amongst women has been linked to impaired fertility, endometriosis and polycystic ovary syndrome, alongside being associated with higher rates of preeclampsia, preterm births, bacterial vaginosis and gestational diabetes, as evidenced by observational studies [16].

Given the nutrients that red meat can provide, it has potential to play an important role in supporting women’s health and general wellbeing. The present publication aims to evaluate inter-relationships between different levels of red meat consumption and women’s micronutrient intakes and status.

## 2. Materials and Methods

This is a secondary analysis of the UK NDNS using data from years 1 to 4 of the rolling programme (2008/2009 to 2011/2012) comparing micronutrient intakes and status of UK female consuming <40 g, 40–69 g and >70 g total red meat daily. In the UK, NDNS total red meat was defined as “beef (g) + burgers (g) + lamb (g) + offal (g) + other Red Meat (g) + pork (g) + processed Red Meat (g) + sausages (g)”. Processed red meat was defined as “manufactured, cured and/or dried meat, including bacon and ham”, as described in Appendix R of NDNS report. Particular focus was given to the percentage of individuals with micronutrient intakes below the LRNI, as this is the level below which deficiencies are most likely to occur.

Two main cut-off levels for total red meat intakes were used: 70 g per day and 40 g per day. A cut-off less than 70 g/day was used based on the Scientific Advisory Committee on Nutrition advice that intakes of red and processed meat (over 90 g/day) should be reduced to less than 70 g/day in order to reduce colorectal cancer risk whilst avoiding having an impact on the proportion of the adults with iron intakes below the LRNI [3].

The less than 40 g per day cut-off was formed based on World Cancer Research Fund (WCRF) guidelines. The WCRF has a “public health goal”, advising that the population average consumption of red meat should be no more than 300 g (11 ounces (oz)) a week with very little, if any, being processed [17]. Based on a 7-day week, this equates to 43 g red meat daily, forming the basis of the 40 g/day cut-off. Given this, the current publication set out to evaluate how these two levels of intake could impact on the micronutrient intakes and status of UK females.

### 2.1. Nutrients of Interest

Nutrition and health claims complying with the European Union’s Nutrition and Health Claims Regulation No. 1924/2006 and the Food Information to Consumer Regulations (EU) No. 1169/2011. European Food Standard Agency stipulations are that for foods to be classified as a ”source” they should contain at least 15% of the Nutrient Reference Value (NRV) and a ”rich source” containing 30% of the NRV. NRVs now replace reference dietary allowances (RDAs) [18]. According to these, beef may be regarded as a rich source of niacin, vitamin B_6_, vitamin B_12_, zinc and source of riboflavin, iron, potassium, phosphorus. Lamb can be categorised as a rich source of niacin, vitamin B12, zinc and source of vitamin B_6_, potassium, phosphorus, pantothenic acid. Finally, pork can be considered a rich source of thiamine, niacin, vitamin B_6_, vitamin B_12_ and source of riboflavin, zinc, potassium, phosphorus, selenium, pantothenic acid [19,20]. Subsequently, the nutrients meeting these claim requirements formed the main focal points in the present article.

### 2.2. National Diet and Nutrition Survey (NDNS)

#### 2.2.1. Subjects and Study Design

The NDNS is a continuous rolling programme throughout the year across the UK (England, Northern Ireland, Wales and Scotland). Full methodological details can be found in Chapter 2 of the full report [21]. In the current analysis, only females aged 11–64 years were included in the secondary analysis.

#### 2.2.2. Socioeconomic Status

Socioeconomic status (SES) was determined through the Index of Multiple Deprivation (IMD), with similar approaches being using in previous NDNS publications [22]. This was divided into five quintiles to provide an estimate regarding the degree of deprivation for each household. 

#### 2.2.3. Micronutrient Data

In the NDNS, participants were asked to keep a record of everything they ate and drank in and outside the home over 4 days including both weekend days in a diary that was provided by the interviewer. Participants were contacted on the second or third day of data collection in person or by telephone to ensure compliance. Average micronutrient intakes were estimated for each individual from the data that were collected throughout the three or four days. Additional information regarding dietary data collection methods can be found in Appendix A of the report. 

In the NDNS during blood collection procedures, a maximum of 35.1 mL of blood was taken from participants aged 16 years and over. Suitably located and resourced field laboratories were used enabling the processing of blood samples within two hours of venepuncture. These had access to a refrigerated centrifuge, piston pipettes and storage facilities at or below −40 °C due to the labile nature of blood indices. Further information about blood sample collecting and processing is provided in Appendix O of the report.

#### 2.2.4. Response Rates

Data in the UK NDNS years 1 to 4 was comprised of 4156 adults, of which 52.6 percent were females. Dietary data was available from 1384 females who completed three or four days of diary data. Blood samples were obtained from 46.3 percent of females included in the NDNS survey. Vitamins B_1_, B_3_ and B_6_ were excluded from the analysis as categorical variables as none of the included females had vitamin B_3_ or B_6_ deficiency and only one female had thiamine deficiency. Data was excluded for non-meat consumers listed as being vegetarian (*n =* 38).

### 2.3. Statistical Analyses

Statistical analyses were performed using IBM Statistical Package for Social Sciences (SPSS), Version 22 (IBM Corp: Armonk, NY, USA) and plots produced using R software. Independent variables were created by organizing data according to daily red meat intake. Plots were constructed to assess normality of the dependent variables. Unpaired *t*-test and Mann–Whitney tests were used to compare the distribution of continuous variables across groups. Chi-Square tests were used to assess the difference in socioeconomic status across groups and the proportions of females with micronutrient intakes below the LRNI.

Sensitivity analysis was performed after excluding vegetarian females (*n =* 38) to ensure that results were not biased. Sensitivity analysis was also performed after excluding females who recorded taking supplements in their diaries (*n* = 302/1384). Multivariate logistic regression was performed to predict the risk of having zinc and iron intake below the LRNI in females consuming <40 g and 40 g to 69 g daily of total red meat. *p*-values were adjusted for age, socioeconomic status, energy intake (kcal), fruit, vegetable and fish intakes and body mass index. A forward approach was used when performing regression analyses where variables were only added if they produced a significant change in the model. *p*-values < 0.001 were considered statistically significant to take into account the multiple testing of dependent variables. Variables were included in multivariate analysis if the *p*-value from the univariate association was less than 0.005.

## 3. Results

### 3.1. Participants

Participant characteristics are presented in Table 1. Data showed that 31.7 percent of females were aged between 11 and 18 years while 68.3 percent were aged 19 years or older. Average body weight was 56.4 kg with 53 percent having a Body Mass Index (BMI) < 25 kg/m^2^ and 47 percent having a BMI ≥ 25 kg/m^2^. SES represented as the IMD was grouped into quintiles. There were no statistically significant differences between quintiles (*p* = 0.607) with respect to meat consumption whether compared by the 40 or 70 cut-off values.

Forty-three percent of UK females had a mean daily red meat intake of less than 40 g whilst 70.5 percent consumed less than 70 g daily. Twenty-seven percent had daily total red meat intakes between 40 g and 69 g daily. Habitual median intake of total red meat was 47 g per day while the median intake for processed red meat was 8.4 g per day. Overall mean daily iron and zinc intakes were below the LRNI in 30.5 and 9.8 percent of participants, respectively. A further 14.7 percent and a quarter (25.1%) had riboflavin and potassium intakes below the LRNI, respectively. Nearly half of the participants (48.5%) had selenium intakes below the LRNI. Mean vitamin D intake for all study participants was 1.98 mg per day. Unfortunately, LRNI data was not available at this time for this nutrient. Mean blood biomarkers were 13.3 mg/dL for hemoglobin, 38.4 mg/dL for ferritin, 13.9 µmol/L for zinc, 256 pmol/L for vitamin B12 and 41.7 nmol/L for 25-hydroxyvitamin D. 

Mean hemoglobin levels exceeded lower limits for hemoglobin set at 120 g/L for non-pregnant female by the World Health Organisation [23]. Plasma ferritin levels were above the lower limit of 15 µ/L below which iron stores are regarded to be depleted and IDA risk increased [23]. Serum vitamin B12 exceeded the lower threshold of the normal range of 150 pmol/L [24]. Mean serum levels of 25-hydroxyvitamin D were above 25 nmol/L which has been used as the lower threshold for vitamin D adequacy below which there is an increased risk of rickets and osteomalacia [25].

### 3.2. Micronutrient Intakes

Data for females consuming less than, or more than 40 and 70 g of total red meat daily were compared (Table 2). Focusing on the 70 g cut-off, those exceeding this were older and had a significantly higher body weight (*p* = 0.001). The percentage of individuals with micronutrients below the LRNI was significantly higher for iron, zinc and potassium amongst those consuming <70 g red meat daily (*p* < 0.0001). Vitamin D intake (LRNI data was not available) was also significantly lower amongst those eating <70 g total red meat daily. No significant differences in nutritional biomarkers were seen.

For the 40 g cut-off, there were no significant differences in age or body weight across groups. The percentage of individuals with micronutrients below the LRNI was significantly higher for iron, zinc, vitamin B12 and potassium amongst those consuming <40 g red meat daily compared with those eating 40 to 68 g daily (*p* < 0.0001). Vitamin D intake was also significantly lower amongst those eating <40 g total red meat daily compared with those eating more. For zinc, the percentage of women falling below the LRNI was substantially higher at 18.9% for those eating <40 g total red meat compared with 2.8% in those eating more than this (Table 2). No significant differences in nutritional biomarkers were found, indicating that greater sample sizes are needed in future NDNS reports to provide adequate statistical power.

Data was also compared against UK females with total red meat intakes <40 g daily and those eating between 40 g and 69 g daily (Table 2). No significant differences were apparent for age or body weight. The proportion of individuals with micronutrients below the LRNI was significantly higher for zinc and vitamin B12 amongst those consuming <40 g total red meat daily compared to those eating between 40 g and 69 g (*p* < 0.001). Iron exhibited the same trend although the association did not reach statistical significance. The proportion of individuals with potassium intake below the LRNI was also higher amongst females consuming <40 g daily, compared with 40 g–69 g daily (31.2 vs. 23.5%, respectively) although it was not significant at the 0.001 level used in this publication. Habitual vitamin D intakes were also significantly lower in this group (*p* < 0.001).

**Figure 1:**
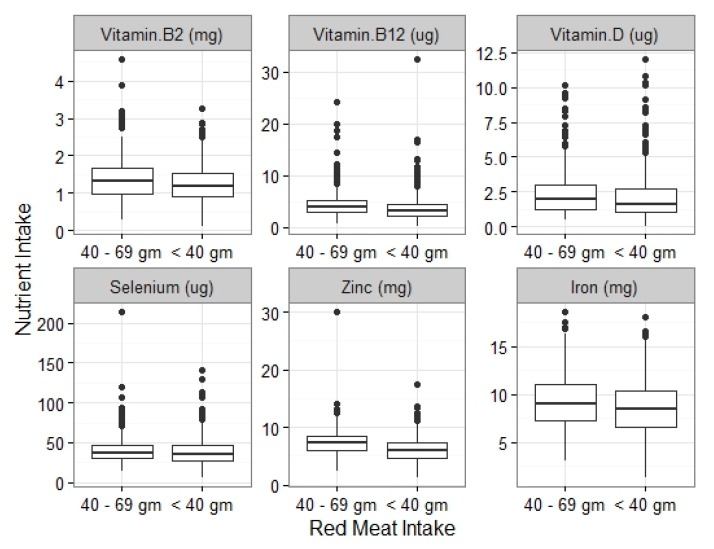
Associations between daily red meat intakes (higher and lower thresholds) and nutrient intakes.

Mean micronutrient intakes were continually lower in females who consumed less than 40 g total red meat daily compared with intakes of 40 g to 69 g daily (Figure 1). Data analysis using Mann–Whitney test showed that mean intakes of iron, zinc, vitamin B_2_, vitamin B_12_, vitamin D and potassium were statistically significantly lower (*p* < 0.001) for UK females ingesting <40 g total red meat daily compared with those having 40 to 69 g daily. Spearman correlations identified a moderate correlation (*r* = 0.51) between zinc intake and total daily red meat intake (*p* < 0.001). There was also a weaker correlation between iron intake and total daily red meat intake (*p* < 0.001, *r* = 0.243). Daily processed red meat intake was not associated with iron intakes in adult women aged 19 to 65 years or girls aged 11 to 18 years. It was, however, significantly associated with zinc intakes in girls aged 11 to 18 and women aged 19 to 65 years and years (*p* < 0.001). 

### 3.3. Regression Modelling

Data adjustments for energy intake (*p* adjusted) showed that the percentage of individuals falling below LRNI for zinc and selenium were significantly higher for both groups with red meat intakes below the 70 g and 40 g cut-offs (*p* < 0.001, Table 2).

Females consuming less than 40 g daily of red meat were four times more likely to have a zinc intake below LRNI (RR 4.6 95% CI (2.28–9.28)) than females consuming 40 g–69 g daily of total red meat (Table 3). The association between red meat intake and iron intake remained non-significant after adjusting for other covariates. Higher energy and fruit and vegetable intakes were also associated with iron and zinc intakes above the LRNI. Women aged 19 to 64 years were also less likely to have zinc and iron intakes below LRNI compared to females aged 11 to 18 years (RR = 0.472 and 0.1, respectively).

### 3.4. Micronutrient Status

Concentrations of nutritional biomarkers were not statistically significantly different between groups (*p* > 0.001 for all analyses) except for ferritin levels which was significant (*p* = 0.002 and *p* = 0.001) for 40 g and 70 g cut-offs, respectively. These results became non-significant after excluding supplement takers (Table 2).

## 4. Discussion

It is well recognised that red meat provides an important range of micronutrients [11]. Recently, the International Agency for Research on Cancer, which forms part of the World Health Organisation classified red meat as ‘probably carcinogenic to humans’ and processed meat as ‘carcinogenic to humans’ [26]. Despite having incomplete insights about underpinning mechanisms [27,28], this advice has overshadowed recognition about the key micronutrients found in red meat [1]. The release of this report has contributed to blanket messages to reduce red meat intakes across all population groups. For example, the recently updated UK Eatwell Guide communicates an overarching message to ‘eat less red and processed meat’ within its main infographic [29].

Recent figures from the UK National Diet and Nutrition Survey (years 5 and 6) clearly demonstrate that intakes of red and processed meat for women aged 19 to 64 years were just 47 g per day; substantially lower than 70 g daily [2,3]. Equally, within the same survey almost half the girls aged 11 to 18 years (48%), and more than a quarter (27%) of females aged 19 to 64 were not reaching the LRNI for iron [2]. The same report showed that a substantial proportion of adults had nutrient intakes below the LRNI for potassium and selenium, with around one-fifth of adults having low blood levels of vitamin D [2].

In 2010, the Scientific Advisory Committee on Nutrition also proposed that “A recommendation to reduce consumption of red and processed meat in order to decrease colorectal cancer risk could have a negative impact on iron and zinc intakes in the UK by increasing the proportion of the population with intakes below the LRNIs for these nutrients” [3]. The present paper found this to be the case, particularly for zinc. Our analysis suggests that UK women consuming <40 g daily total red meat were four times more likely to have zinc intakes below LRNI compared to those eating between 40 g and 69 g daily. Equally, the present analysis identified that eating less than 40 g red meat daily significantly reduced UK women’s vitamin D intakes from dietary sources. 

It should be considered that zinc is an important nutrient for women of reproductive age. This can be obtained successfully from dietary sources including red meat [30] provided that these are consumed in adequate amounts. Interestingly, recent work has identified a significant relationship between serum zinc levels and symptoms of premenstrual syndrome amongst a sample of 48 young girls [31]. Amongst pre-menopausal women zinc has been linked to improved cognitive and emotional functioning [32]. Equally, it has recently been estimated that addressing vitamin D inadequacy in pregnant women in England and Wales could reduce the number of cases of pre-eclampsia by 4126; and would result in a net saving of £18.6 million for the National Health Service (NHS) in England and Wales [33]. Whilst supplements have their own role to play, addressing the diet to include balanced amounts of lean red meat is another way forward in terms of helping to reduce the impact of these micronutrient shortfalls.

No statistically significant differences in blood biomarkers were found between groups when different levels of red meat intake were compared. One explanation could be that only a sub-sample of participants from the NDNS provided blood samples which could have weakened the statistical power of blood analyte data. When comparing blood analytes against blood biomarker cut-offs, these were above cut-offs applied in the NDNS and typically used to indicate deficiencies or shortfalls. These results indicate that the LRNI may not be reflective of nutritional deficiencies. However, it should also be considered that different blood biomarker cut-offs would also yield different findings. For example, the Institute of Medicine regards a level of 50 nmol/L as being sufficient to optimize bone health in the majority of the population [34]. Had this cut-off been used (rather than 25 nmol/L) mean blood levels would have been below this across all groups. 

Future studies should also consider measuring biomarkers of dietary meat intake such as urea, creatine, creatinine, carnitine, carnosine, anserine, opidine-1 and 3-methylhistidine to accurately reflect and validate habitually reported meat intakes [35] as mis-reporting could have occurred which is a limitation of all methods of measuring food intakes in dietary surveys [2]. It is also a shortcoming that this was a secondary analysis, rather than a primary study outcome. Future studies should also look to differentiate between lean red meat and processed red meat. It would also be of interest for impending studies to assess specific markers of women’s health.

## 5. Conclusions

In conclusion, the present paper found that consuming less than 40 g total red meat daily could have adverse implications on UK women’s zinc and vitamin D intakes in particular. Clearly, more remains to be known about the nutritional implications of diets with a lower red meat content. However, this work emphasizes that before blanket recommendations can be put into place advising the public to reduce red meat intakes, the wider nutritional significance of these should be considered. This particularly applies to women who already have some of the lowest red meat intakes. 

## Figures and Tables

**Table 1:** Characteristics of study subjects (*n* = 1384).

Age		LRNI ^a^
11–18 years (*n*, %)	439 (31.7%)	--
19–64 years (*n*, %)	945 (68.3%)	--
Weight (kg) (*n* = 1301)	56.4 (56.4–77.8)	--
BMI (kg/m^2^) (*n =* 1296)		--
Less than 25 kg/m^2^	687 (53%)	--
Equal or More than 25 kg/m^2^	609 (47%)	--
SES (IMD Score)		--
0.53–8.49	245 (21.3%)	--
8.49–13.79	225 (19.6%)	--
13.79–21.35	212 (18.5%)	--
21.35–34.17	230 (20%)	--
34.17–87.8	237 (20.6%)	--
Fe < LRNI (*n*, %)	422 (30.5%)	4.7 mg/day M; 8.0 mg/day F
Zn < LRNI (*n*, %)	135 (9.8%)	5.5 mg/day M; 4.0 mg/day F
Vit. B1 < LRNI (*n*, %)	1 (0.1%)	0.23 mg/1000 kcal
Vit. B2 < LRNI (*n*, %)	203 (14.7%)	0.8 mg/day
Vit. B12 < LRNI (*n*, %)	23 (1.7%)	1.0 μg/day
Se < LRNI (*n*, %)	671 (48.5%)	40 μg/day
K < LRNI (*n*, %)	348 (25.1%)	2000 mg/day
Vit. D intake (mg)	1.98 (1.29–3.11)	--
Hemoglobin (mg/dL) (*n =* 444)	13.3 (12.6–13.8)	--
Plasma Ferritin (mg/dL) (*n =* 450)	34.86 (19.15–69.23)	--
Plasma Zn (μmol/L)	13.97 (12.46–15.55)	--
Serum vit. B12 (pmol/L) (*n =* 446)	256 (204–312.25)	--
Serum 25-hydroxyvitamin D (nmol/L) (*n* = 445)	41.65 (26.68–59.43)	--

Key: LRNI, Lower Reference Nutrient Intake; BMI, Body Mass Index; SES, Socioeconomic status; IMD, Index of Multiple Deprivation, ^a^ Department of Health (DH, 1991) Dietary Reference Values for Food Energy and Nutrients for the United Kingdom. HMSO: London. Data is shown as count (percentage) for categorical variables and as Median (IQR) for continuous variables. Supplement takers were excluded.

**Table 2:** Different red meat intakes in relation to micronutrient intakes and status in UK females.

	Red Meat < 70 (*n =* 976)	Red Meat ≥ 70 (*n =* 408)	*P* Univariate	Adjusted *P* ^b^	Red Meat < 40 (*n =* 597)	Red Meat ≥ 40 (*n =* 787)	*P* Univariate	Adjusted *P* ^b^	Red Meat 40–69 g (*n =* 379)	*P* Univariate (Compared against < 40)	Adjusted *P* ^b^
*Demographics.*											
Age											
11–18 years	332 (34%)	107 (26.2%)	0.005	----	210 (35.2%)	229 (29.1%)	0.016	---	122 (32.2%)	0.373	--
19–64 years	664 (66%)	301 (73.8%)			387 (64.8%)	558 (70.9%)			257 (67.8%)		
Weight (kg)	64.7 (55.7–75.6)	67 [57.4–81.8]	0.001 *	---	63.9 [55.4–75.5]	66.2 [57.2–79.1]	0.007	---	67.6 (15.9)	0.285	--
*Micronutrient intakes.*											
Fe < LRNI	340 (34.8%)	82 (20.1%)	<0.001 *	0.166	225 (37.7%)	197 (25%)	<0.001 *	0.357	115 (30.3%)	0.019	
Zn < LRNI	131 (13.4%)	4 (1%)	<0.001 *	<0.001 *	113 (18.9%)	22 (2.8%)	<0.001 *	<0.001 *	18 (4.7%)	<0.001 *	<0.001 *
Vit. B2 < LRNI	159 (16.3%)	44 (10.8%)	0.008	0.829	107 (17.9%)	96 (12.2%)	0.003	0.2	107 (17.9%)	0.083	
Vit. B12 < LRNI	23 (2.4%)	0 (0%)	0.002	0.99	22 (3.7%)	1 (0.1%)	<0.001 *	0.991	1 (0.3%)	<0.001 *	0.99
K < LRNI	275 (28.2%)	73 (17.9%)	<0.001 *	0.592	186 (31.2%)	162 (20.6%)	<0.001 *	0.156	89 (23.5%)	0.009	
Se < LRNI	497 (50.9%)	174 (42.6%)	0.005	<0.001 *	306 (51.3%)	365 (46.4%)	0.072	<0.001 *	191 (50.4%)	0.973	
Vit. D intake (mg)	1.74 (1.1–2.85)	2.26 (1.63–3.31)	<0.001 *	<0.001 *	1.6 (1–2.74)	2.12 (1.47–3.18)	<0.001 *	<0.001 *	1.98 (1.2–3)	<0.001 *	<0.001 *
*Blood analytes a.*											
Hemoglobin (g/dL)	13.2 (12.6–13.8)	13.3 (12.9–13.9)	0.193	---	13.2 (12.6–13.8)	13.3 (12.8–13.9)	0.296	---	13.3 (12.6–13.8)	0.361	--
Ferritin (µg/L)	31.1 (18.5–61.5)	45.4 (23–79.6)	0.014	---	29.3 (16.4–55.9)	40.3 (20.6–72.6)	0.065	---	33.2 (19.7–66.6)	0.109	--
Zn (µmol/L)	14.1 (12.5–14.1)	13.9 (12.5–15.5)	0.572	---	14.3 (12.6–15.6)	13.9 (12.2–15.3)	0.289	---	13.8 (12.5–15.6)	0.454	--
Vit. B12 (pmol/L)	256 (198–314)	256 (211–303)	0.776	---	258 (198–311)	256 (206–316)	0.876	---	254 (195–327)	0.907	--
Vit. D (nmol/L)	40.2 (25.3–58.4)	44.5 (29.7–62.6)	0.075	---	41.1 (27.9–58.4)	42.1 (26.3–61.4)	0.844	---	39.2 (22.8–58.3)	0.386	--

Data is shown as count (%) and mean/SD or median (IQR) for continuous variables. *p*-values < 0.001 were considered statistically significant. ^a^ Results shown for blood analytes after the exclusion of females who recorded taking supplements. ^b^ Adjusted for energy intake, Age, SES, fruit and vegetable intake, fish intake, white meat intake and body mass index. * *p*-values < 0.001 were considered statistically significant.

**Table 3:** Logistic regression models to predict iron and zinc intake below lower RNI (*n* = 764).

	Iron Intake below LRNI		Zinc Intake below LRNI	
	Model *R^2^* Total = 0.518		Model *R^2^* Total = 0.544	
Independent Variable	Relative Risk (95% CI)	*p*	Relative Risk (95% CI)	*p*
Daily Red meat intake (<40 g daily compared to 40–69 g)	----	---	4.6 (2.28–9.28)	<0.001 *
Total fruits and vegetables	0.992 (0.99–0.994)	<0.001 *	0.995 (0.993–0.998)	<0.001 *
Total white meat intake	---	---	---	---
Total fish intake	---	---	---	---
Age (adults)	0.472 (0.311–0.716)	<0.001 *	0.1 (0.052–0.194)	<0.001 *
BMI (<25 kg/m^2^)	----	---	---	---
QIMD	---	---	---	---
Energy intake (Kcal/day)	0.996 (0.996–0.997)	<0.001 *	0.995 (0.994–0.996)	<0.001 *

Variables used in the model: BMI, quintile of index of multiple deprivation (QIMD), total red meat, total fish, total white meat, total fruit and vegetable intake and energy intake (Kcal). Red meat intake, body mass index, age and SES QIMD were included as categorical variables. Remaining variables were included as continuous variables. * *p*-values < 0.001 were considered statistically significant.
